# Switching of delta opioid receptor subtypes in central amygdala microcircuits is associated with anxiety states in pain

**DOI:** 10.1016/j.jbc.2021.100277

**Published:** 2021-01-09

**Authors:** Wenjie Zhou, Yanhua Li, Xiaojing Meng, An Liu, Yu Mao, Xia Zhu, Qian Meng, Yan Jin, Zhi Zhang, Wenjuan Tao

**Affiliations:** 1 Hefei National Laboratory for Physical Sciences at the Microscale, Department of Biophysics and Neurobiology, University of Science and Technology of China, Hefei, China; 2 Department of Science and Education, Affiliated Psychological Hospital of Anhui Medical University, Hefei, China; 3 Department of Physiology, School of Basic Medical Sciences, Anhui Medical University, Hefei, China

**Keywords:** chronic pain, delta opioid receptor (DOR), anxiety, central amygdala (CeA), basolateral amygdala (BLA), parabrachial nucleus (PBN), ACSF, artificial cerebrospinal fluid, BLA, basolateral amygdala, BNTX, 7-benzylidenenaltrexone, CeA, central amygdala, CFA, complete Freund’s adjuvant, DOR, delta opioid receptor, EPM, elevated plus maze, GSH, glutathione, mEPSC, miniature excitatory postsynaptic current, MOR, μ-opioid receptor, NMDG, N-methyl-D-glucamine, NTB, naltriben, OFT, open field test, PBN, parabrachial nucleus, PFA, paraformaldehyde, PTX, picrotoxin

## Abstract

Anxiety is often comorbid with pain. Delta opioid receptors (DORs) are promising targets for the treatment of pain and mental disorders with little addictive potential. However, their roles in anxiety symptoms at different stages of pain are unclear. In the current study, mice with inflammatory pain at the fourth hour following complete Freund’s adjuvant (CFA) injection displayed significant anxiety-like behavior, which disappeared at the seventh day. Combining electrophysiology, optogenetics, and pharmacology, we found that activation of delta opioid receptor 1 (DOR1) in the central nucleus amygdala (CeA) inhibited both the anxiolytic excitatory input from the basolateral amygdala (BLA) and the anxiogenic excitatory input from the parabrachial nucleus (PBN). In contrast, activation of delta opioid receptor 2 (DOR2) did not affect CeA excitatory synaptic transmission in normal and 4-h CFA mice but inhibited the excitatory projection from the PBN rather than the BLA in 7-day CFA mice. Furthermore, the function of both DOR1 and DOR2 was downregulated to the point of not being detectable in the CeA of mice at the 21st day following CFA injection. Taken together, these results suggest that functional switching of DOR1 and DOR2 is associated with anxiety states at different stages of pain *via* modulating the activity of specific pathways (BLA-CeA and PBN-CeA).

Pain is a complex disorder including an unpleasant sensory and emotional experience. Pain is closely associated with a number of physiological and psychological maladaptations, including anxiety (1, 2), which may lead to an excessive duration and intensity of pain (3). However, the mechanism underlying comorbid anxiety during the development of persistent pain is not fully understood.

Opioid drugs are most often prescribed for pain control but cause several severe side effects after repeated use, including analgesic tolerance and addiction (4). Opioid receptors belong to the G-protein-coupled receptors, which have three subtypes: the μ-opioid receptor (MOR), δ-opioid receptor (DOR), and κ-opioid receptor (5). Both the analgesic and rewarding effects of opioid drugs are primarily mediated by MORs (6). In contrast, pharmacological and genetic data highlight DOR agonists as promising targets for treating pain with limited side effects (7, 8, 9). DORs also play a crucial role in emotion processing (10). DOR knockout mice exhibit increased emotional disorders and pain sensations (11, 12), and selective activation of DOR using the agonist SNC80 reduces anxiety- and depression-like behaviors (13, 14). Even though only one DOR gene has been identified until now, two subtypes of DOR have been pharmacologically identified: DOR1, which is sensitive to DPDPE and antagonized by 7-benzylidenenaltrexone (BNTX), and DOR2, which is sensitive to deltorphin II and antagonized by naltriben (NTB) (15). Activation of either DOR1 or DOR2 in the ventromedial medulla increases the pain threshold (16). However, only the DOR2 selective antagonist abolishes the anxiolytic effects of novel DOR agonist KNT-127 (1,2,3,4,4a,5,12,12a-octahydro-2-methyl-4aβ,1β-([1,2]benzenomethano)-2,6-diazanaphthacene-12aβ,17-diol) (17). A recent study found that both DOR1 and DOR2 coexist in the same neurons and produce the opposite responses (18). These data suggest that the two DOR subtypes in the central nervous system may have different functions. However, the role of the two DORs in pain-associated anxiety remains unclear.

The DORs are widely expressed in the central nervous system, including the hippocampus and hypothalamus, as well as the basal ganglia and amygdala (19, 20). The amygdala has been well recognized in pain and anxiety modulation (21); in particular, the central amygdala (CeA), referred to as the “nociceptive amygdala” (21), serves as the output of the amygdala. The opioidergic system of the CeA is involved in anxiety-related behaviors (22). However, the function of different subtypes of DORs in the CeA for pain-associated anxiety is not clear. In this study, we aimed to characterize the role of the two DOR subtypes (DOR1 and DOR2) in the CeA for pain-associated anxiety at different stages of pain in a mouse model that was established by complete Freund’s adjuvant (CFA).

## Results

### CFA 4 h mice rather than CFA 7-day mice displayed anxiety-like behaviors

**Figure 1:**
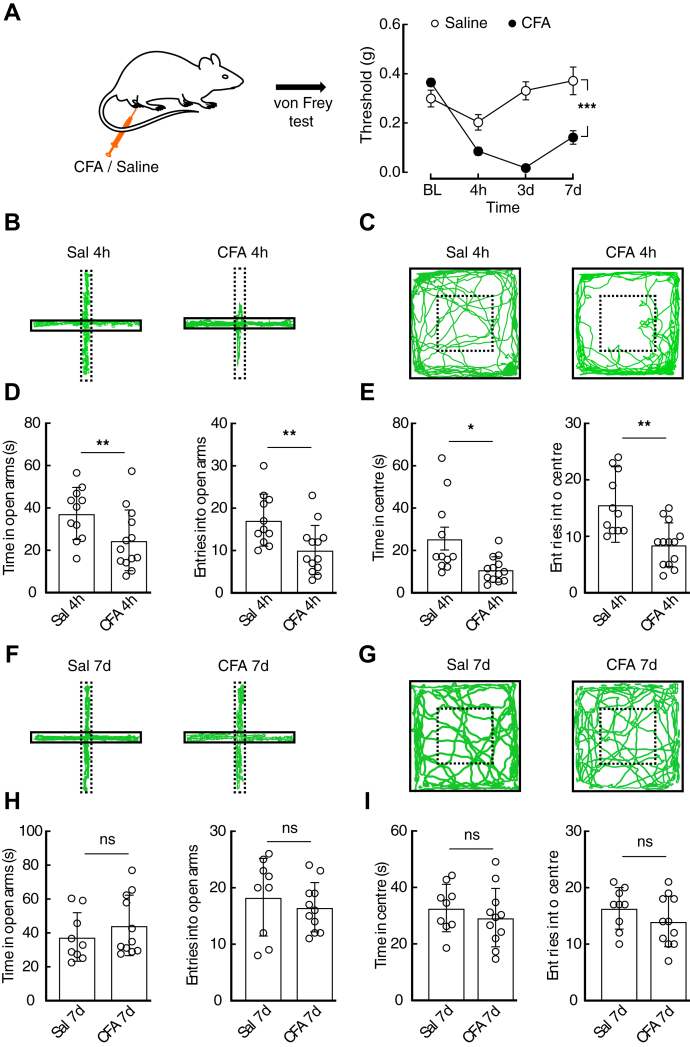
**CFA 4 h rather than CFA 7-day mice display anxiety-like behaviors.***A*, schematic of CFA (*n* = 7 mice) or saline (*n* = 6 mice) injection (*left*) and the time course of hyperalgesia (*right*) measured by the von Frey test (time × group interaction, *F*_3,33_ = 14.74, *p* < 0.0001). *B* and *C*, locomotion traces of CFA 4-h and saline 4-h (Sal 4 h) mice in the elevated plus maze test (EPM, *B*) and open field test (OFT, *C*). *D* and *E*, summarized data of the time spent in and entries into the open arms of the EPM (*D*, Sal 4 h, *n* = 11 mice; CFA 4 h, *n* = 13 mice; time: *t*_22_ = 2.31, *p* = 0.0307; entries: *t*_22_ = 2.882, *p* = 0.0086) and central area of the OFT (*E*, time: *t*_22_ = 2.654, *p* = 0.0145; entries: *t*_22_ = 3.765, *p* = 0.0011) from the indicated groups. *F* and *G*, locomotion traces of CFA 7-day and saline 7-day (Sal 7 days) mice in the EPM test (*F*) or OFT (*G*). *H* and *I*, summarized data of the time spent in or entries into the open arms of the EPM (*H*, time: *t*_18_ = 0.9284, *p* = 0.3655; entries: *t*_18_ = 0.7077, *p* = 0.4882) or the central area of the OFT (*I*, time: *t*_18_ = 0.7922, *p* = 0.4386; entries: *t*_18_ = 1.255, *p* = 0.2255) in the mice treated with saline (*n* = 9 mice) or CFA (*n* = 11 mice) for 7 days. Significance was assessed by two-way repeated measures (RM) ANOVA with *post hoc* comparison between groups in *A* and a two-tailed unpaired Student’s *t*-test in *D*, *E*, *H*, and *I*. The data are expressed as the mean ± SD. ∗*p* < 0.05; ∗∗*p* < 0.01; ∗∗∗*p* < 0.001. ns, no significance.

Anxiety-like behaviors in pain have been well documented in animal models (23, 24). Here, anxiety-like behaviors were assessed at different time points of inflammatory pain. Following CFA injection, the mechanical pain threshold was significantly decreased (Fig. 1*A*). Then, anxiety-like behaviors of the mice treated with CFA for 4 h (CFA 4 h) or 7 days (CFA 7 days) were separately assessed by the elevated plus maze (EPM) test and open field test (OFT). Interestingly, the CFA 4 h mice displayed anxiety-like behaviors, indicated by less time spent in and fewer entries into the open arms of the EPM or central area of the open field compared with the saline 4-h mice (Fig. 1, *B*–*E*), but no significant difference was detected between saline 7-day (Sal 7 days) and CFA 7-day mice (Fig. 1, *F*–*I*). Moreover, no significant difference in the total traveled distance was detected in CFA 4-h or CFA 7-day mice compared with the control mice (Fig. S1).

### Inhibitory action of DOR1 on the increased excitatory transmission in CFA 4 h mice was replaced by that of DOR2 in CFA 7-day mice

**Figure 2:**
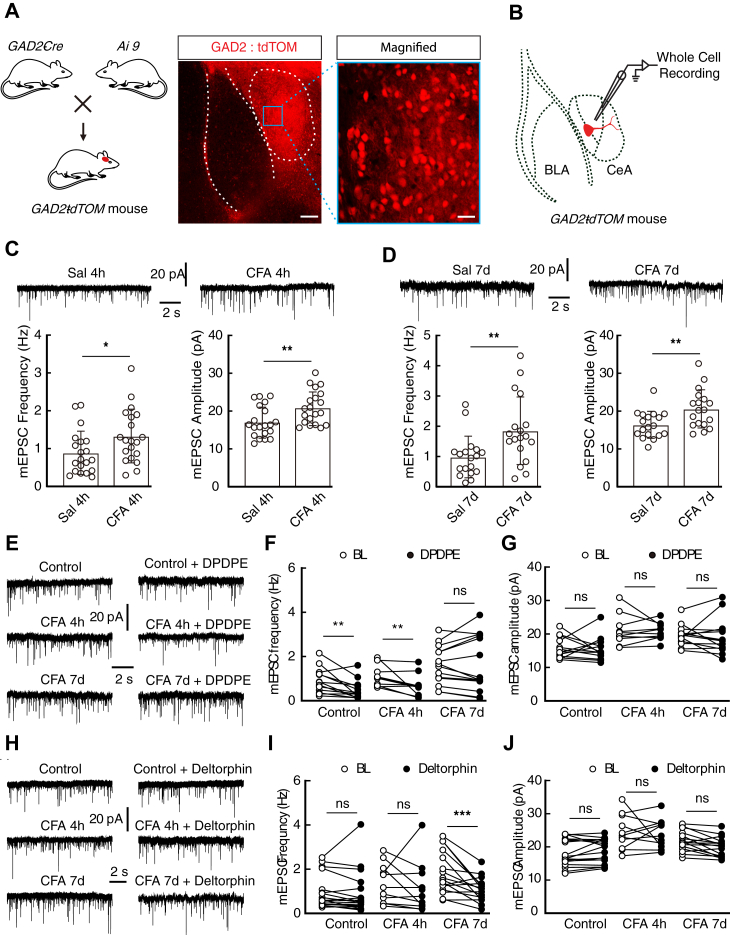
**Inflammatory pain increases the excitatory transmission in the CeA.***A*, schematic of crossing *Gad2-Cre* mice and *Ai9* reporter mice (*left*) and representative images of tdTomato-expressing GABAergic neurons in the CeA from *GAD2-tdTOM* mice (*righ*t). Scale bars, 200 μm left, 50 μm right. *B*, schematic of recording configuration in acute brain slices. *C*, representative traces and the statistical data of the mEPSC recorded in saline 4 h (Sal 4 h, *n* = 20 cells from four mice) and CFA 4 h mice (*n* = 20 cells from 4 mice; frequency: *t*_38_ = 2.17, *p* = 0.0364; amplitude: *t*_38_ = 2.824, *p* = 0.0075). *D*, representative traces and statistical data of the mEPSC recorded from the CeA neurons of 7-day saline (Sal 7 days, *n* = 17 cells from three mice) and CFA 7-day mice (*n* = 17 cells from four mice; frequency: *t*_34_ = 2.802; amplitude: *p* = 0.0083). *E*, representative traces of the mEPSC recorded in the CeA GABAergic neurons from normal, CFA 4 h and CFA 7-day mice without or with DPDPE (1 μM). *F* and *G*, summarized data of the frequency (*F*) and amplitude (*G*) of the mEPSC recorded in the CeA GABA neurons of normal (*n* = 13 cells from three mice; frequency: *t*_12_ = 3.229, *p* = 0.0072; amplitude: *t*_12_ = 0.2756, *p* = 0.7875), CFA 4 h (*n* = 10 cells from three mice; frequency: *t*_9_ = 2.667, *p* = 0.0258; amplitude: *t*_9_ = 0.2472, *p* = 0.8103), and CFA 7-day mice (*n* = 14 cells from four mice; frequency: *t*_13_ = 0.3035, *p* = 0.7663; amplitude: *t*_13_ = 0.3943, *p* = 0.6998) without or with DPDPE. *H*, typical traces of the mEPSC recorded from normal, CFA 4 h, and CFA 7-day mice without or with deltorphin II (1 μM). *I* and *J*, statistical data of the frequency (*I*) and amplitude (*J*) of the mEPSC recorded without or with deltorphin II in the CeA of normal (*n* = 18 cells from four mice; frequency: *t*_17_ = 0.4815, *p* = 0.6363; amplitude: *t*_17_ = 1.149, *p* = 0.2663), CFA 4 h (*n* = 11 cells from three mice; frequency: *t*_10_ = 0.7549, *p* = 0.4677; amplitude: *t*_10_ = 0.2902, *p* = 0.7776), and CFA 7-day mice (*n* = 16 cells from four mice; frequency: *t*_15_ = 4.076, *p* = 0.001; amplitude: *t*_15_ = 1.781, *p* = 0.0951) without or with deltorphin II. Significance was assessed by a two-tailed unpaired Student’s *t*-test in *C* and *D,* a two-tailed paired Student’s *t* test in *F*, *G*, *I*, and *J*. The data are expressed as the mean ± SD. ∗*p* < 0.05; ∗∗*p* < 0.01; ∗∗∗*p* < 0.001. ns, no significance.

We next determined how the anxiety-like behaviors induced by acute pain disappeared during the development of persistent pain. Considering that the CeA consists of approximately 95% GABA neurons (25), we crossed *glutamic acid decarboxylase 2* (GAD2, a GABA synthetic enzyme)-*Cre* mice with *Ai9* (RCL-tdT) mice (26) to produce transgenic mice with red tdTomato-expressing GABA (*GAD2-tdTOM*) neurons for visualized whole-cell patch-clamp recording in acute brain slices (Fig. 2, *A* and *B*). The excitatory transmission in the CeA has been shown to be involved in pain and emotional processing (27, 28). Through whole-cell recording, we found that both the frequency and the amplitude of the miniature excitatory postsynaptic currents (mEPSC) recorded in the CeA from CFA 4 h mice were significantly increased compared with those from saline mice (Fig. 2*C*). Similarly, both the frequency and the amplitude of the mEPSC recorded in CFA 7-day mice were increased (Fig. 2*D*).

DORs are evolving targets for the treatment of pain and mental disorders (10) and could be recruited onto the cell membrane in a chronic pain state (29). Thus, we determined whether the increased excitatory transmission in the CeA was affected by DORs. We found that following perfusion of DPDPE, the frequency of the mEPSC was significantly reduced in normal and CFA 4 h mice without affecting the amplitude (Fig. 2, *E*–*G*). However, the increased frequency and amplitude of the mEPSC recorded in CFA 7-day mice were unaffected (Fig. 2, *E*–*G*). Following perfusion of deltorphin II, both the frequency and the amplitude of the mEPSC recorded in normal and CFA 4 h mice were unaffected (Fig. 2, *H*–*J*), but the increased frequency recorded in CFA 7-day mice was significantly inhibited without affecting the amplitude (Fig. 2, *H*–*J*). These data suggested that the inhibitory action of DOR1 on the excitatory transmission was switched on by that of DOR2 during the development of pain.

### The anxiolytic pathway from the basolateral amygdala (BLA) to the CeA was unaffected by DOR activation in CFA 7-day mice

**Figure 3:**
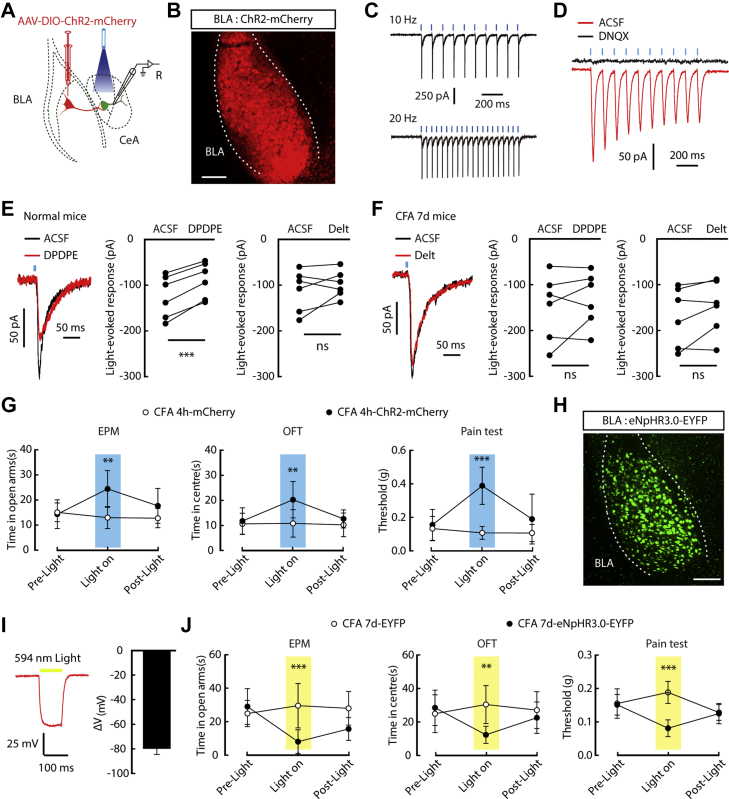
**Disappearance of DOR1 from the excitatory presynaptic terminals from the BLA in CFA 7-day mice.***A*, schematic of AAV-DIO-ChR2-mCherry injected into the BLA of *CaMKII-Cre* mice and recording configuration performed in acute slices. *B*, representative image of DIO-ChR2-mCherry expression in the BLA of *CaMKII-Cre* mice. Scale bar, 200 μm. *C*, representative traces of action potentials evoked by 473 nm light recorded from a ChR2-mCherry-expressing neuron. *D*, sample traces of light-evoked synaptic response recorded from a CeA neuron following photostimulation of BLA^Glu^ terminals without or with DNQX. *E*, sample traces and statistical data showing the effect of DPDPE (*n* = 3 mice; *t*_5_ = 8.84, *p* = 0.0003) or deltorphin II (*n* = 3 mice; Delt, *t*_5_ = 1.215, *p* = 0.2787) on the light-evoked EPSCs recorded in CeA neurons from normal mice. *F*, sample traces and statistical data showing the effect of DPDPE (*t*_5_ = 0.5238, *p* = 0.6228) or deltorphin II (*t*_5_ = 1.901, *p* = 0.1158) on the light-evoked EPSCs (*n* = 3 mice) from CFA 7-day mice. *G*, summarized data showing the effect of optical activation of BLA^Glu^ terminals in the CeA of CFA 4 h *CaMKII-Cre* mice in the EPM (*left*, *n* = 6 mice each group; time × group interaction, *F*_2,20_ = 4.931, *p* = 0.0182), OFT (*middle*, *n* = 7 mice each group; time × group interaction, *F*_2,24_ = 5.063, *p* = 0.0146), and mechanical pain test (*right*, CFA 4 h-ChR2-mCherry, *n* = 7 mice; CFA 4 h-mCherry, *n* = 9 mice; time × group interaction, *F*_2,28_ = 13.8, *p* < 0.0001). *H*, Expression of DIO-eNpHR3.0-EYFP in the BLA of *CaMKII-Cre* mice. Scale bar, 200 μm. *I*, representative trace (*left*) and summarized data (*right*) of the hyperpolarized membrane potential recorded from eNpHR3.0-EYFP-expressing neurons following photostimulation (*n* = 9 cells). *J*, summarized data showing the effect of optical inhibition of the BLA^Glu^-CeA pathway in CFA 7-day *CaMKII-Cre* mice in the EPM (*left*, CFA 7-day-eNpHR3.0-EYFP, *n* = 8 mice; CFA 7-day-EYFP, *n* = 6 mice; time × group interaction, *F*_2,24_ = 5.694, *p* = 0.0095), OFT (*middle*, time × group interaction, *F*_2,24_ = 7.605, *p* = 0.0028), and mechanical pain test (time × group interaction, *F*_2,24_ = 4.427, *p* = 0.0229). Significance was assessed by a two-tailed paired Student’s *t*-test in *E* and *F*, and two-way RM ANOVA with *post hoc* comparison between groups in *G* and *J*. The data are expressed as the mean ± SD. ∗∗*p* < 0.01; ∗∗∗*p* < 0.001. ns, no significance.

Given that different excitatory inputs to the CeA have different functions (30, 31), we determined whether dysfunction of DOR1 or the emergence of DOR2 was involved in affecting a specific pathway. It has been shown that there are increased excitatory inputs from the BLA to the CeA in pain states (27). To investigate the effects of DOR activation on the excitatory input from the BLA to the CeA, adeno-associated virus expressing Cre-dependent channelrhodopsin-2 (AAV-DIO-ChR2-mCherry) was injected into the BLA of *CaMKII-Cre* mice, a mouse line that drives *Cre* enzyme expression in excitatory neurons, and whole-cell recording was performed on CeA neurons (Fig. 3, *A*–*C*). The glutamate-mediated EPSCs, which could be blocked by AMPA receptor antagonist 6,7-dinitroquinoxaline-2,3-dione (DNQX), were recorded in CeA neurons following optical activation of ChR2-containing terminals (Fig. 3*D*). Interestingly, the recorded light-evoked EPSCs were inhibited by perfusion of DPDPE rather than deltorphin II in normal and CFA 4 h mice (Fig. 3*E* and Fig. S2*A*). However, the light-evoked EPSCs were unaffected by perfusion of both DPDPE and deltorphin II in CFA 7-day mice (Fig. 3*F*).

Then, we examined the roles of the BLA^Glu^-CeA pathway in pain and anxiety. In normal *CaMKII-Cre* mice, the pain threshold was increased after optical activation of ChR2-containing fibers in the CeA (Fig. S2*B*). The anxiety-like behaviors and hyperalgesia observed in CFA 4 h mice were alleviated following optical activation of this pathway (Fig. 3*G*). Similarly, in CFA 7-day mice, the pain threshold, the time spent in the center of the open field, and the time spent in the open arms of the EPM were increased by the optical manipulation as well (Fig. S2*E*). Furthermore, we injected an adeno-associated virus expressing Cre-dependent eNpHR3.0-EYFP (AAV-DIO-eNpHR3.0-EYFP) into the BLA of *CaMKII-Cre* mice to inhibit the BLA-CeA pathway (Fig. 3*H* and Fig. S2*D*). The BLA neurons expressing eNpHR3.0-EYFP were hyperpolarized by 594 nm light (Fig. 3*I*). After optical inhibition of eNpHR3.0-containing fibers in the CeA, the pain sensitization and anxiety-like behaviors of CFA 4 h mice were deteriorated (Fig. S2*F*); CFA 7-day mice displayed anxiety-like behaviors (Fig. 3*J*).

### The anxiogenic pathway from the parabrachial nucleus (PBN) to the CeA was inhibited by DOR2 activation in CFA 7-day mice

**Figure 4:**
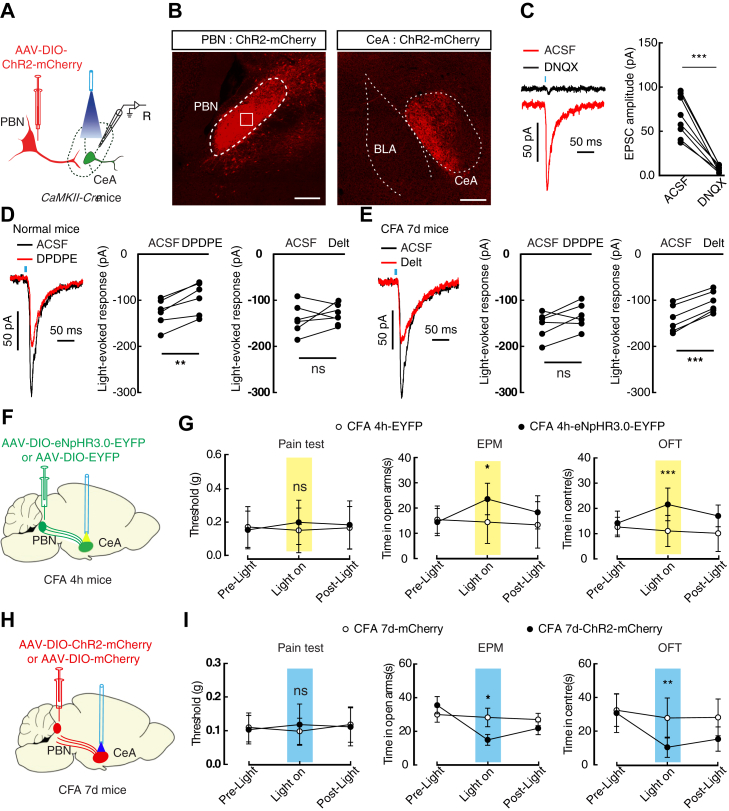
**Emergence of DOR2 on the excitatory presynaptic terminals from the PBN in CFA 7-days mice.***A*, schematic of the virus injection and recording configuration. *B*, representative images of AAV-DIO-ChR2-mCherry expression in the PBN of *CaMKII-Cre* mice and the mCherry^+^ fibers observed in the CeA. Scale bars, 200 μm. *C*, sample traces and statistical chart of light-evoked synaptic responses recorded from CeA neurons after optical activation of the PBN terminals without or with DNQX (*n* = 8 cells from three mice). *D*, sample traces and statistical data showing the effect of DPDPE (*n* = 3 mice; *t*_5_ = 5.714, *p* = 0.0023) or deltorphin II (Delt, *n* = 3 mice; *t*_5_ = 1.011, *p* = 0.3584) on the light-evoked EPSCs of normal mice following optical activation of BLA^Glu^ terminals. *E*, sample traces and statistical data showing the effect of deltorphin II (Delt, *n* = 3 mice; *t*_5_ = 12.23, *p* < 0.0001) or DPDPE (*n* = 3 mice; *t*_5_ = 1.949, *p* = 0.1088) on the light-evoked EPSCs of CFA 7-day mice following optical activation of BLA^Glu^ terminals. *F*, schematic of viral injection and optical inhibition of the PBN-CeA pathway in CFA 4 h *CaMKII-Cre* mice. *G*, performance of CFA 4 h *CaMKII-Cre* mice following optical inhibition of PBN^Glu^-CeA in the EPM (*left*, *n* = 9 mice each group; time × group interaction, *F*_2,32_ = 3.657, *p* = 0.0371), OFT (*middle*, time × group interaction, *F*_2,32_ = 5.336, *p* = 0.01), and mechanical pain test (*right*, CFA 4 h-eNpHR3.0-EYFP, *n* = 7 mice; CFA 4 h-EYFP, *n* = 6 mice; time × group interaction, *F*_2,24_ = 0.2008, *p* = 0.8194). *H*, schematic of viral injection and optical activation of the PBN-CeA pathway in CFA 7-day *CaMKII-Cre* mice. *I*, performance of CFA 7-day *CaMKII-Cre* mice following optical activation of the PBN^Glu^-CeA pathway in the EPM (*left*, *n* = 9 mice each group; time × group interaction, *F*_2,32_ = 3.972, *p* = 0.0288), OFT (*middle*, time × group interaction, *F*_2,32_ = 4.2, *p* = 0.024), and mechanical pain test (CFA 7-day-ChR2-mCherry, *n* = 7 mice; CFA 7-day-mCherry, *n* = 6 mice; time × group interaction, *F*_2,22_ = 0.05693, *p* = 0.9448). Significance was assessed by a two-tailed paired Student’s *t*-test in *C*–*E*, and two-way RM ANOVA with *post hoc* comparison between groups in *G* and *I*. The data are expressed as the mean ± SD. ∗*p* < 0.05; ∗∗*p* < 0.01; ∗∗∗*p* < 0.001. ns, no significance.

In addition to the BLA, the increased excitatory input from the PBN to the CeA in pain states has also been reported (30, 32, 33). We next examined whether the excitatory input from the PBN could be modulated by DORs. First, adeno-associated virus expressing DIO-ChR2-mCherry was injected into the PBN of *CaMKII-Cre* mice, and robust mCherry fibers were observed in the CeA (Fig. 4, *A* and *B*). After optical activation of ChR2-containing fibers in the CeA, light-evoked EPSCs were reliably induced in CeA neurons and were blocked by perfusion of DNQX (Fig. 4*C*). In normal and CFA 4 h mice, we found that the light-evoked EPSCs were inhibited by perfusion of DPDPE rather than deltorphin II (Fig. 4*D* and Fig. S3*A*). In contrast, the light-evoked EPSCs recorded in CFA 7-day mice were inhibited by perfusion of deltorphin II rather than DPDPE (Fig. 4*E*).

We then determined whether the PBN^Glu^-CeA pathway was involved in pain and anxiety-like behaviors. The Cre-dependent eNpHR3.0-EYFP-expressing virus was injected into the PBN of *CaMKII-Cre* mice, and the optical fiber was implanted toward the CeA to selectively inhibit the PBN-CeA pathway (Fig. 4*F*). We found that in CFA 4 h mice and CFA 7-day mice, the pain threshold was not affected, but the time spent in the center of the open field or open arms of the EPM was increased following optical inhibition of the PBN-CeA pathway (Fig. 4*G* and Fig. S3*B*). In addition, following optical activation of ChR2-containing PBN^Glu^ fibers in the CeA, the anxiety-like behaviors of CFA 4 h mice were increased, and CFA 7-day mice displayed anxiety-like behaviors (Fig. 4, *H* and *I* and Fig. S3*C*). These data suggest that the anxiogenic PBN^Glu^-CeA pathway is likely inhibited by DOR2 activation in CFA 7-day mice.

### Anxiolytic role of DOR2 activation in CFA 7-day mice

**Figure 5:**
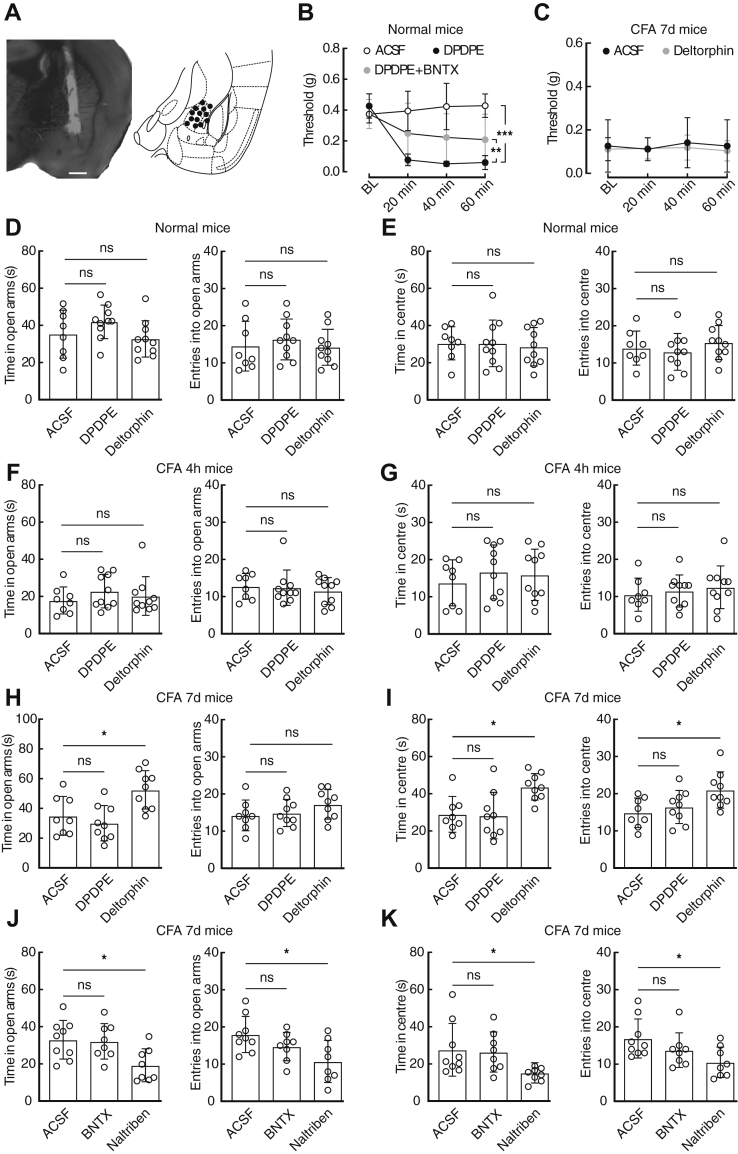
**Activation of DOR2 in the CeA produces anxiolytic effects.***A*, representative image (*left*) and drawing of cannula injection sites (*right*). Scale bar, 500 μm. *B*, pain threshold of the normal mice following the CeA injection of ACSF (*n* = 7 mice), DPDPE (*n* = 7 mice), and DPDPE + BNTX (*n* = 8 mice; time × group interaction, *F*_6,57_ = 5.312, *p* = 0.0002). *C*, pain threshold of CFA 7-day mice following the CeA injection of ACSF (*n* = 8 mice) and deltorphin II (*n* = 8 mice; time × group interaction, *F*_3,42_ = 0.09595, *p* = 0.9619). *D* and *E*, summarized data of normal mice in the EPM (*D*, time, *F*_2,25_ = 2.013, *p* = 0.1546; entries, *F*_2,25_ = 0.3989, *p* = 0.6753) and OFT (*E*, time, *F*_2,25_ = 0.087, *p* = 0.9173; entries, *F*_2,25_ = 0.71, *p* = 0.5013) following injection of ACSF (*n* = 8 mice), DPDPE (*n* = 10 mice), and deltorphin II (*n* = 10 mice) into the CeA. *F* and *G*, summarized data of CFA 4 h mice in the EPM (*F*, time, *F*_2,25_ = 0.6836, *p* = 0.514; entries, *F*_2,25_ = 0.2371, *p* = 0.7907) and OFT (*G*, time, *F*_2,25_ = 0.4282, *p* = 0.6564; entries, *F*_2,25_ = 0.3689, *p* = 0.6952) following injection of ACSF (*n* = 8 mice), DPDPE (*n* = 10 mice), and deltorphin II (*n* = 10 mice) into the CeA. *H* and *I*, performance of CFA 7-day mice in the EPM (*H*, time, *F*_2,23_ = 7.794, *p* = 0.0026; entries, *F*_2,23_ = 1.391, *p* = 0.2691) and OFT (*I*, time, *F*_2,23_ = 6.745, *p* = 0.005; entries, *F*_2,23_ = 4.393, *p* = 0.0242) following injection of ACSF (*n* = 8 mice), DPDPE (*n* = 9 mice), and deltorphin II (*n* = 9 mice) into the CeA. *J* and *K*, performance of CFA 7-day mice in the EPM (*J*, time: *F*_2,22_ = 5.18, *p* = 0.0143; entries: *F*_2,22_ = 4.777, *p* = 0.0189) and OFT (*K*, time: *F*_2,22_ = 4.117, *p* = 0.0303; entries: *F*_2,22_ = 3.896, *p* = 0.0356) following injection of ACSF (*n* = 9 mice), BNTX (*n* = 8 mice), and naltriben (*n* = 8 mice) into the CeA. Significance was assessed by two-way RM ANOVA with *post hoc* comparison between groups in *B* and *C*, ordinary one-way ANOVA with *post hoc* comparison between groups in *D*–*K*. The data are expressed as the mean ± SD. ∗*p* < 0.05; ∗∗*p* < 0.01, ∗∗∗*p* < 0.001. ns, no significance.

To determine the role of the dysfunction of DOR1 and the emergence of DOR2 in the CeA, DPDPE and deltorphin II were separately microinjected into the CeA (Fig. 5*A*). Following CeA infusion of DPDPE, the pain threshold of normal mice was significantly decreased, which could be partially reversed by preinfusion of the antagonist BNTX (Fig. 5*B*). This difference was not observed in either CFA 4 h mice or CFA 7-day mice (Fig. S4*A*). In addition, the performance of normal, CFA 4 h, and CFA 7-day mice in the EPM test and OFT was not significantly affected following CeA infusion of DPDPE (Fig. 5, *D*–*I*). In contrast, CeA infusion of deltorphin II had no effects on the pain threshold of normal, CFA 4-h, or CFA 7-day mice (Fig. 5*C* and Fig. S4*B*), while the time spent in the center of the open field or open arms of the EPM was increased for CFA 7-day mice but not for normal or CFA 4 h mice (Fig. 5, *D*–*I*). Of note, CeA infusion of naltriben rather than BNTX in CFA 7-day mice induced anxiety-like behaviors (Fig. 5, *J* and *K*). These data suggest that the emergence of DOR2 in the CeA is likely involved in the disappearance of anxiety-like behavior in CFA 7-day mice.

### Dysfunction of DORs in CFA 21-day mice

**Figure 6:**
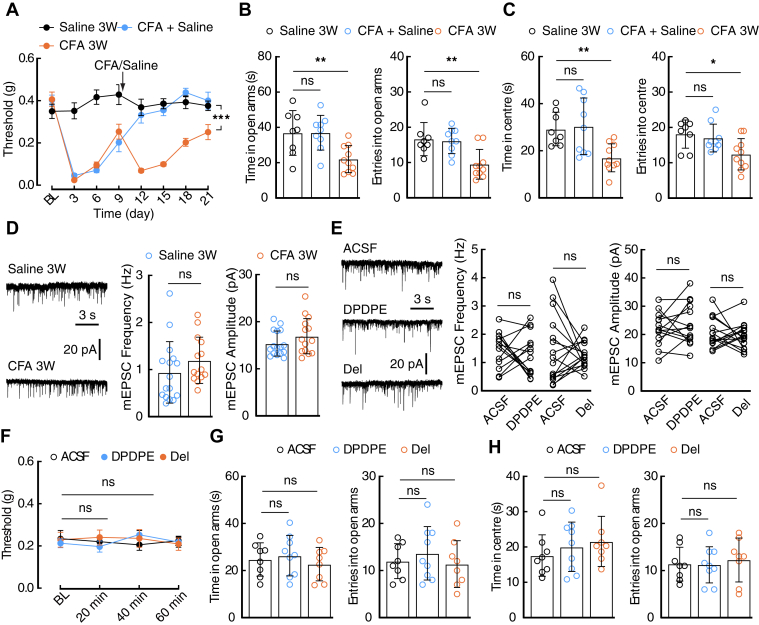
**Disappearance of DORs in the CeA of chronic pain mice.***A*, time course of hyperalgesia induced by a second injection of CFA (saline, *n* = 8 mice; CFA+Saline, *n* = 10 mice; CFA, *n* = 10 mice; time × group interaction, *F*_14,175_ = 13.17, *p* < 0.0001). *B* and *C*, performance of saline 3W (*n* = 8 mice), CFA+Saline (*n* = 9 mice), and CFA 3W (*n* = 10 mice) mice in the EPM (*B*, time: *F*_2,24_ = 6.937, *p* = 0.0042; entries: *F*_2,24_ = 8.536, *p* = 0.0016) and OFT (*C*, time: *F*_2,24_ = 6.884, *p* = 0.0043; entries: *F*_2,24_ = 4.99, *p* = 0.0154). *D*, representative traces and the statistical data of the mEPSC recorded in 21 day-saline mice (*n* = 16 cells from three mice) and 21-day CFA mice (*n* = 14 cells from three mice; frequency: *t*_28_ = 1.197, *p* = 0.2412; amplitude: *t*_28_ = 1.558, *p* = 0.1305). *E*, typical traces and statistical data of the mEPSC recorded without or with DPDPE (*n* = 14 cells from four mice; frequency: *t*_13_ = 0.6846, *p* = 0.5056; amplitude: *t*_13_ = 0.8845, *p* = 0.3925) or deltorphin II (*n* = 16 cells from five mice; frequency: *t*_15_ = 0.5532, *p* = 0.5883; amplitude: *t*_15_ = 0.4942, *p* = 0.6283). *F*, pain threshold of the CFA 3W mice following the CeA injection of ACSF (*n* = 7 mice), DPDPE (*n* = 7 mice) or deltorphin II (*n* = 8 mice; *F*_6,57_ = 0.3483, *p* = 0.908). *G* and *H*, statistical data of the CFA 3W mice in EPM (*G*, time: *F*_2,22_ = 0.4611, *p* = 0.6366; entries: *F*_2,22_ = 0.5046, *p* = 0.6105) or OFT (*H*, time: *F*_2,22_ = 0.7259, *p* = 0.4951; entries: *F*_2,22_ = 0.1554, *p* = 0.857) following CeA infusion of ACSF (*n* = 8 mice), DPDPE (*n* = 9 mice) and deltorphin II (*n* = 8 mice). Significance was assessed by two-way RM ANOVA with *post hoc* comparison between groups in *A* and *F*, and a two-tailed unpaired Student’s *t* test in *D*, a two-tailed paired Student’s *t*-test in *E*, and ordinary one-way ANOVA in *B*, *C*, *G*, and *H*. The data are expressed as the mean ± SD. ∗*p* < 0.05; ∗∗*p* < 0.01, ∗∗∗*p* < 0.001. ns, no significance.

**Figure 7:**
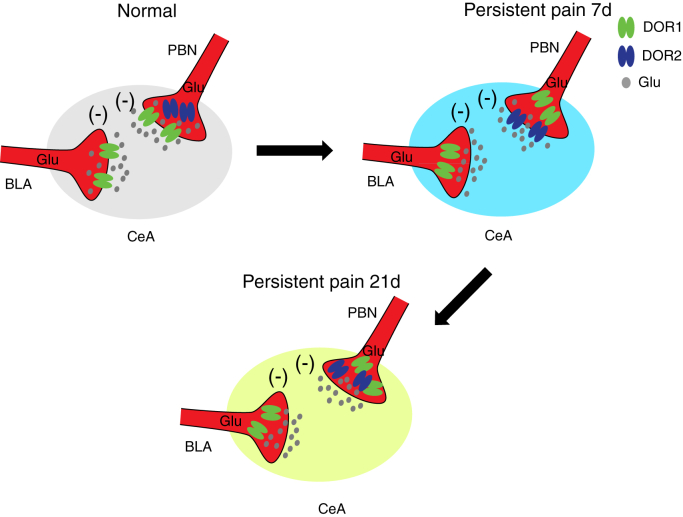
**A model for the switching of DOR1 and DOR2 during the development of persistent pain.** In normal mice, DOR1 was expressed on the excitatory presynaptic terminals from the BLA and the PBN. During the development of pain (inflammatory pain, 7 days), DOR1 disappeared from the excitatory presynaptic terminals from both the BLA and the PBN, and DOR2 emerged specifically on the excitatory presynaptic terminals from the PBN. Both of these changes likely contributed to the alleviation of anxiety-like behaviors induced by acute pain. In 21-day inflammatory pain mice with anxiety-like behaviors, both DORs had disappeared from the excitatory presynapse.

Anxiety-associated behaviors have frequently been observed in clinical patients and animals with chronic pain (23, 34). Consistent with previous reports (23, 35), we found that mice treated with CFA for 21 days (CFA 21 days) displayed anxiety-like behaviors (Fig. 6, *A*–*C*); the mEPSC recorded in these mice was slightly increased, which was not affected by perfusion of DPDPE or deltorphin II (Fig. 6, *D* and *E*). In addition, CeA infusion of DPDPE or deltorphin II had no effects on the pain sensitization or anxiety-like behaviors of CFA 21-day mice (Fig. 6, *F*–*H*). Overall, these results indicate that the switching and dysfunction of DOR1 and DOR2 subtypes are associated with anxiety states at different stages of inflammatory pain (Fig. 7).

## Discussion

Pain and anxiety are frequently encountered together in the clinic (23, 36). Here, we found that anxiety-like behaviors were reliably induced by acute pain (CFA 4 h) and disappeared during the development of persistent pain (CFA 7-day). At CFA 21 days, anxiety-like behaviors reoccurred. Further empirical research shows that the switching of different subtypes of DORs (DOR1 and DOR2) in the CeA is associated with anxiety states at different stages of pain. Central to these processes is that activation of DOR1, but not DOR2, may contribute to CFA 4 h-induced anxiety-like behaviors. In contrast, the membrane trafficking of DOR2 and dysfunction of DOR1 at CFA 7 days cause the disappearance of anxiety-like behaviors at this time.

It has been shown that pain stimuli can induce the membrane trafficking of DORs, activation of which by endogenous opioid systems in turn relieves the pain (29). In this study, we identified the function of DOR1 and DOR2 in pain-associated anxiety-like behaviors using pharmacological experiments with electrophysiological and behavioral approaches. Owing to the limitation of the selective antibodies for immunostaining of different subtypes of DORs, classical definition of DOR subtypes is based on pharmacological effects by selective DOR agonists, such as DPDPE and deltorphin II (37). Pharmacological studies suggest that at least two DOR subtypes are expressed in the central nervous system, which are relevant to anxiety and pain (17), but only one DOR gene has been cloned (19). Based on the inability of certain DOR antagonists to block the effect of DOR agonists, DOR1 is classified as the receptors activated by DPDPE and sensitive to BNTX, while DOR2 is activated by deltorphin II and sensitive to naltriben (38). Because DPDPE is rapidly degraded by peptidases, whereas deltorphin II has a much longer-lasting effect, as it is resistant to these peptidases, they also display different effects on behavioral phenotypes in experimental research (39, 40). For example, in a 55 °C warm water tail-flick test, a single injection of DPDPE produced antinociception within 30 min, while deltorphin II effects could last for about 1 h with same dose (41). Based on our results on the pharmacological effects of selective DOR agonists on mEPSCs, pain, and anxiety-like behaviors, we propose that DOR1 is responsible for acute inflammatory pain-associated anxiogenic behavior but DOR2 is not due to a lack of functional expression of this protein at this time point. Meanwhile, DOR2 is trafficked to the neuronal membrane in a persistent pain state (CFA 7 days), activation of which blocks anxiety-like behaviors. Owing to the importance of biochemical and immunostaining evidence for the expression of DOR1 and DOR2 on the neuronal membrane or synapses, these relationships are clearly worthy of further investigation.

The selectivity and pharmacokinetics of different DOR agonists may play impactful roles in behavioral phenotypes. Notably, DPDPE is not as selective as deltorphin II for DORs and can activate MOR; the response is equi-efficacious and equipotent in DOR, MOR-DOR, and κ-opioid (KOR)-DOR heteromers in cell lines (37, 42). This raises the possibility that activation of MORs is involved in the effects of DPDPE at CFA 4 h. In addition, numerous studies have proposed that DOR and MOR or KOR oligomerize to form a heteromer, which also probably results in the pharmacological subtypes of DORs (43, 44). Indeed, MOR and KOR are highly expressed in the CeA (45). The behavioral differences between DOR1 and DOR2 subtypes in normal and CFA 7-day mice likely represent phenotypic receptors that display different sensitivities to the selective agonists (46, 47). Another possible explanation for the lacking function of DOR1 or MOR at CFA 7 days according to the deficient actions of DPDPE at this time point is the desensitization of DORs to their agonists, which is a common occurrence in multiple environments, *e.g.*, persistent activation of the endogenous opioid system and persistent pain (48).

The diversity of CeA GABAergic neurons is also a possible reason that DPDPE and deltorphin II have different effects on excitatory transmission. Specifically, the frequency of mEPSCs from only a portion of the CeA GABAergic neurons from CFA 4 h mice was decreased following perfusion of deltorphin II. The molecular identity and topographical location of CeA GABAergic neurons are physiologically, genetically, and functionally heterogeneous (33, 49, 50). Different classes of CeA GABAergic neurons, including neurons expressing corticotropin-releasing hormone (CRH), somatostatin (SOM), protein kinase C- δ (PKC-δ), and neurotensin (51, 52, 53), may have distinct responses and roles in pain (50). For example, inflammatory pain was found to decrease the excitability of regular spiking periaqueductal gray (PAG)-projecting CeA GABAergic neurons without affecting the bursting PAG-projecting neurons (54). In a nerve-injured mouse model, the PKC-δ neurons were sensitized, driving hyperalgesia, but the excitability of SOM neurons was inhibited, driving hypoalgesia (50). Meanwhile, CeA CRH neurons exerted pro- or antinociceptive effects depending on the type of CRH receptors (55), which is well known to be related to anxiety-like behaviors (56). These findings suggest that different types of GABAergic neurons play distinct or even opposing roles in the development of pain and anxiety. Given that CeA GABAergic neurons are highly heterogeneous, differential distribution of DOR1 and DOR2 in different subtypes of GABAergic neurons may contribute to these processes.

The CeA accepts broad excitatory inputs from the whole brain implicated in mediating different physiological functions (32). The opposing roles of the BLA^Glu^-CeA pathway and the PBN^Glu^-CeA pathway in anxiety and fear learning have been identified (28, 30, 32). However, how these two pathways are regulated during the development of persistent pain remains elusive. Previous studies reported that the endogenous opioid system is activated by pain stimuli (57). Specifically, the amount of β-endorphin in the CeA is increased by noxious stimulation (58). If so, activation of DOR1 on BLA^Glu^ terminals in the CeA by endogenous opioids would produce anxiety in CFA 4 h mice. In addition, we found that the BLA^Glu^-CeA pathway was unaffected by DOR1 agonists in CFA 7-day mice, and the PBN^Glu^-CeA pathway was inhibited by DOR2 agonists. We thus propose that the dysfunction of DOR1 from the BLA^Glu^ terminal at CFA 7 days may decrease its inhibition of the BLA-CeA pathway, which subsequently prevents the anxiety state at this time point. The emergence of functional DOR2 on PBN^Glu^ terminals in the CeA at CFA 7 days, which may be activated by endogenous opioids, would further exert anxiolytic effects. Therefore, both our study and previous studies suggest that the induction of membrane trafficking of DOR2 and its selective agonists would be a promising strategy for treating anxiety (17).

## Experimental procedures

### Animals and inflammatory pain model

During the experiment, male C57, *CaMKII-Cre*, *Ai9* (RCL-tdT) (26), and *GAD2-Cre* (purchased from Charles River or Jackson Laboratories) mice aged 8 to 10 weeks were used. The mice were housed five per cage under a 12-h light/dark cycle with stable temperature (23–25 °C) and *ad libitum* access to water and food. Under deep isoflurane anesthesia,CFA (10 μl, Sigma-Aldrich) was injected into the plantar of the left hind paws of the mice using an insulin syringe (BD Corp) to induce persistent inflammatory pain, and saline was injected as control. The pain threshold was measured by von Frey test as we previously reported (59). Considering that the stress effects that may be caused during the von Frey pain test procedure could contaminate our results, different cohorts of mice were used for the tests of pain and anxiety-like behaviors. All animal protocols were approved by Animal Care and Use Committee of the University of Science and Technology of China.

### Virus injection

The virus was injected as previously reported (34). Briefly, the mice were fixed on a stereotactic frame (RWD, Shenzhen, China) under deeply anesthetize using isoflurane (5% for induction; 1.5–2% for maintenance). A heating pad was used to maintain the core body temperature of the animals at 36 °C. For activation of PBN-CeA pathway, the Cre-dependent virus rAAV-Ef1α-DIO-hChR2 (H134R)-mCherry-WPRE-pA (AAV-DIO-ChR2-mCherry, AAV2/9, 1.63 × 10^13^ vg/ml, 200 nl) was injected into the PBN (AP, −5.25 mm; ML, −1.25 mm; DV, −2.50 mm) of *CaMKII-Cre* mice using calibrated glass microelectrodes connected to an infusion pump (micro 4, WPI, USA, 30 nl/min). For optical inhibition of PBN-CeA pathway, the Cre-dependent virus rAAV-Ef1α-DIO-eNpHR3.0-EYFP-WPRE-pA (AAV-DIO-eNpHR3.0-EYFP, AAV2/9, 1.18 × 10^13^ vg/ml) was injected into the PBN of *CaMKII-Cre* mice. The rAAV-Ef1α-DIO-mCherry-WPRE-pA (AAV-DIO-mCherry, AAV2/8, 8.93 ×10^12^ vg/ml, 200 nl), or rAAV-DIO-EYFP-WPRE-pA (AAV-DIO-EYFP, AAV2/9, 1.95 × 10^12^ vg/ml) virus was injected as control. The same viruses were injected into the BLA (AP, −1.25 mm; ML, −3.10 mm; DV, −4.20 mm) of *CaMKII-Cre* mice for the optical activation or inhibition of BLA-CeA pathway. Following the virus injection, the optical fiber (diameter, 200 μm, Newdoon, China) was chronically implanted into the ipsilateral CeA (AP, −1.15; ML, −2.65 mm; DV, −4.05 mm) using dental cement. Unless otherwise stated, all viruses were packaged by BrainVTA.

### Immunofluorescence

After the experiment, the mice were deeply anesthetized with pentobarbital sodium (50 mg/kg, i.p.) and sequentially perfused with saline and 4% (w/v) paraformaldehyde (PFA). The brains were subsequently removed and postfixed in 4% PFA at 4 °C overnight. After cryoprotection of the brains with 30% (w/v) sucrose, coronal sections (40 μm) were cut on a cryostat (Leica CM1860) and used for immunofluorescence. Fluorescence signals expressed by the virus were visualized using a Leica DM2500 camera and Zeiss LSM710 microscope.

### Behavior test and optogenetics manipulation

For the behavior test, the mice were transported into the testing room 1 day prior to testing for habitation. During the testing session, the movement trajectories were recorded and subsequently analyzed offline using EthoVision XT software (Noldus). The entries into and time spent in the center of open field or the open arms of the elevated plus maze were counted. For the optogenetics manipulation, the mice were anesthetized using isoflurane and the implanted fibers were connected to a laser generator (Shanghai Fiblaser) using optical fiber sleeves. The blue light (473 nm, 1–3 mW, 15 ms pulses, 20 Hz) or yellow light (594 nm, 5–8 mW, constant) was controlled by Master-8 pulse stimulator (A.M.P.I.), which also applied in the control group. The mice were subjected to light epochs of 5 min OFF/5 min ON/5 min OFF. The location of the fibers was examined after all of the experiments, and data obtained from mice in which the fibers were outside the desired brain region were discarded.

#### Open field test

The open field apparatus that consists a square area (25 cm × 25 cm) and a marginal area (50 cm × 50 cm × 60 cm) was used to assay the anxiety. The mice were placed in one corner of the apparatus and allowed to move freely. The movement of the mice was recorded, and the number of entries into and the amount of time spent in the central area were calculated offline. The apparatus was cleaned using 75% ethanol between each test to remove olfactory cues.

#### Elevated plus maze test

The apparatus consists of a central platform (6 × 6 cm), two closed arms (30 × 6 × 20 cm), and two opposing open arms (30 × 6 cm). The maze was placed about 100 cm above the floor. During the testing, the mice were placed in the central platform facing a closed arm and allowed to explore the maze. The movement trajectories were analyzed offline, and the time spent in the open arms and the number of entries into the open arms were calculated after recording. The apparatus was cleaned between tests using 75% ethanol.

### Whole-cell recordings

#### Brain slices preparation

The mice were deeply anesthetized with 2% pentobarbital sodium and then intracardially perfused with ∼20 ml ice-cold oxygenated N-methyl-D-glucamine (NMDG) artificial cerebrospinal fluid (ACSF) containing (in mM): 93 NMDG, 2.5 KCl, 1.2 NaH_2_PO_4_, 30 NaHCO_3_, 20 HEPES, 25 glucose, 2 thiourea, 5 Na-ascorbate, 3 Na-pyruvate, 0.5 CaCl_2_, 10 MgSO_4_, and 3 glutathione (GSH). The PH was adjusted to 7.3 to 7.4 and the osmolarity was adjusted to 300 to 305 mOsm/kg. After perfusion, the brain slices containing CeA was quickly sectioned by vibrating microtome (VT1200s, Leica, Germany). Then the slices were transiently incubated in oxygenated NMDG ACSF at 33 °C for 10 to 12 min and subsequently incubated in N-2-hydroxyethylpiperazine-N-2-ethanesulfonic acid (HEPES) ACSF containing (in mM) 92 NaCl, 2.5 KCl, 1.2 NaH_2_PO_4_, 30 NaHCO_3_, 20 HEPES, 25 glucose, 2 thiourea, 5 Na-ascorbate, 3 Na-pyruvate, 2 CaCl_2_, 2 MgSO_4_, and 3 glutathione (GSH) that pH adjusted to 7.3 to 7.4 and osmolarity adjusted to 300 to 305 mOsm/kg for at least 1 h at 28 °C. After that, the slices were used for whole-cell recording.

#### Whole-cell recordings

The brain slices were then transferred to the chamber (Warner Instruments) that continuously perfused with oxygenated standard ACSF containing (in mM) 124 NaCl, 2.4 CaCl_2_, 5 KCl, 1.3 MgSO_4_, 26.2 NaHCO_3_, 1.2 KH_2_PO_4_ and 10 glucose (pH: 7.3–7.4 osmolarity: 300–305 mOsm/kg) for recording at 33 °C that maintained by solution heater (TC-344B, Warner Instruments). For the recording of miniature excitatory postsynaptic current (mEPSC), the tetrodotoxin (TTX, 1 μM) was added with picrotoxin (PTX, 50 μM) into the standard ACSF. The recording pipettes was pulled from borosilicate glass capillaries (VitalSense Scientific Instruments Co, Ltd) using horizontal puller (P1000, Sutter Instruments) and filled with intracellular solution containing (in mM): 130 K-gluconate, 2 MgCl_2_, 5 KCl, 0.6 EGTA, 10 HEPES, 2 Mg-ATP and 0.3 Na-GTP (osmolarity: 285–290 mOsm/kg, pH: 7.2). During the recordings, the neurons were held at −70 mV using voltage clamp model, and the signals were acquired using Multiclamp 700B amplifier and filtered at 2.8 kHz, digitized at 10 kHz. The recording will be terminated if the series resistance changed more than 20% during the recording. The data was offline analyzed using Clampfit 10.7 software (Molecular Devices). To assay the effect of the DORs agonist on the synaptic transmission, about 10 min baseline was recorded followed by perfusion of DPDPE (1 μm) or deltorphin II (1 μm) for 5 min. To test the light-evoked response from the BLA or PBN to CeA, the optical stimulation was delivered using a laser (Shanghai Fiblaser Technology Co, Ltd) through a 200 μm optical fiber during the whole-cell recording.

### Local drug infusion

First, the cannula was inserted toward the CeA and fixed on the skull with dental cement. The mice were allowed to recover for 7 days before drug infusion. During the infusion, the mice were anesthetized with isoflurane, and the drugs (0.3 nmol DPDPE, 0.1 nmol naltriben, 1 pmol BNTX, and 0.05 nmol deltorphin II) were infused through the injector and controlled infusion pump *via* polyethylene tubing (16). All drugs were dissolved in ACSF and injected in a volume of 0.2 μl at a rate of 0.2 μl/min. The standard ACSF was infused as control. The anxiety-like behaviors were assayed 30 min after the drug infusion. All of the drugs used for pharmacology were purchased from Tocris.

### Statistical analysis

We conducted simple statistical comparisons using Student’s *t*-test. ANOVA (one-way and two-way) and *post hoc* analyses were used to statistically analyze the data from the experimental groups with multiple comparisons. GraphPad Prism 7 (GraphPad Software, Inc) was used for the statistical analyses and graphing. All data are expressed as the mean ± SD, and significance levels are indicated as ∗*p* < 0.05, ∗∗*p* < 0.01, ∗∗∗*p* < 0.001.

## Data availability

The data that support the findings of this study are all contained in the results section of the article and also available upon request. For requests, please contact Wenjuan Tao at wjtao01@ahmu.edu.cn.

## Conflict of interest

The authors declare no conflicts of interest in regard to this manuscript.
