# Supplementary material for: Switching of delta opioid receptor subtypes in central amygdala microcircuits is associated with anxiety states in pain
Source: J Biol Chem. 2021 Jan 9;296:100277. doi: 10.1016/j.jbc.2021.100277 (PMC7948800; doi:10.1016/j.jbc.2021.100277)
Supplement: Supplemental Figures S1–S4 [file mmc1.pdf]

Supporting Information for
Switching of delta opioid receptor subtypes in central amygdala microcircuits is associated with anxiety states in pain

Wenjie Zhou[2†], Yanhua Li[2†], Xiaojing Meng[3], An Liu[1], Yu Mao[1, 2], Xia Zhu[2], Qian Meng[2], Yan Jin[2], Zhi Zhang[2]* and Wenjuan Tao[1, 2]*

[1]School of Basic Medical Sciences, Anhui Medical University, Meishan Road 81, Hefei 230022, China.
[2]Hefei National Laboratory for Physical Sciences at the Microscale, Department of Biophysics and Neurobiology, University of Science and Technology of China, Hefei 230027, PR China.
[3]Department of Science and Education, Affiliated Psychological Hospital of Anhui Medical University, Hefei, PR China.
†These authors contributed equally to this work.

* Corresponding author: Wenjuan Tao
E-mail: wjtao01@ahmu.edu.cn
Zhi Zhang
E-mail: zhizhang@ustc.edu.cn

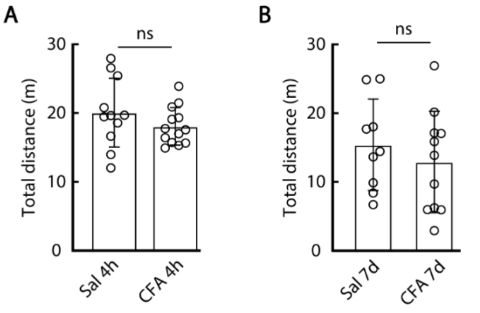

Figure S1. Travelled distance of CFA 4h and CFA 7d mice in the OFT. (**A**, **B**) Summarized data of movement distance of CFA 4h (**A**, Sal 4h, *n* = 11 mice; CFA 4h, *n* = 13 mice; t_{22} = 1.237, *p* = 0.2292), CFA 7d mice (**B**, Sal 7d, *n* = 9 mice; CFA 7d, *n* = 11 mice; t_{18} = 0.7944, *p* = 0.4373), and the control mice in the OFT. Significance was assessed by two-tailed unpaired Student's *t* test. The data are expressed as the mean ± SD. ns, no significance.

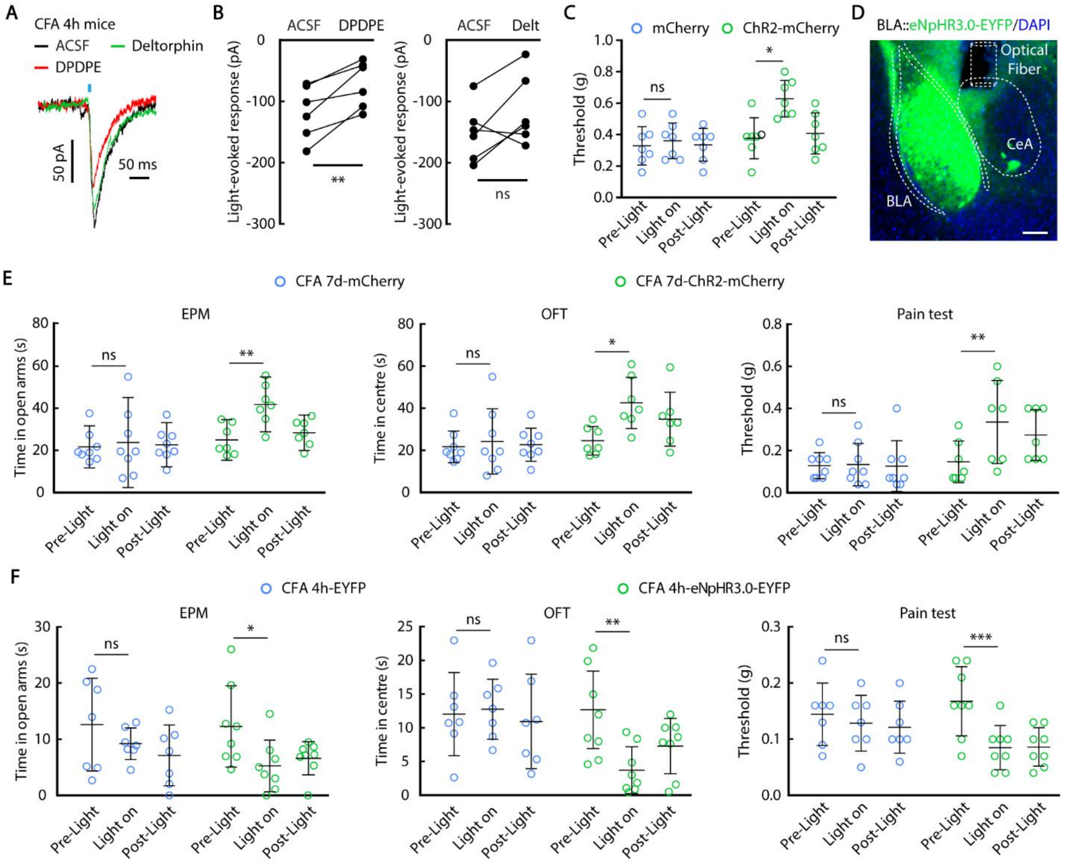

Figure S2. Optical inhibition of the BLA-CeA pathway deteriorates the anxiety of CFA 4h mice. (**A**, **B**) Sample traces (**A**) and statistical data (**B**) showing the effect of DPDPE (n = 6 cells from three mice; $t_5 = 4.356$, $p = 0.0073$) or deltorphin II (n = 6 cells from three mice; $t_5 = 1.832$, $p = 0.1265$) on the light-evoked EPSCs following optical activation of BLA^{Glu} terminals in the CeA from CFA 4h mice. (**C**) Pain threshold of normal *CaMKII-Cre* mice with the BLA infusion of AAV-DIO-ChR2-mCherry (ChR2-mCherry) or control virus (mCherry) after 473nm light was delivered in the CeA (n = 7 mice each group; time × group interaction, $F_{2,24} = 3.58$, $p = 0.0436$). (**D**) Typical image of the optic fiber site in the CeA. Scale bar, 200 µm. (**E**) Summarized data showing the effect of optical activation of BLA^{Glu} terminals in the CeA of CFA 7d *CaMKII-Cre* mice in the EPM (left, CFA 7d-mCherry, n = 8 mice; CFA 7d-ChR2-mCherry, n = 7 mice; time × group interaction, $F_{2,26} = 5.344$, $p = 0.0114$), OFT (middle, time × group interaction, $F_{2,26} = 3.544$, $p = 0.0436$) and pain threshold (right, time × group interaction, $F_{2,26} = 3.498$, $p = 0.0452$). (**F**) Summarized data showing the effect of optical inhibition of BLA^{Glu} terminals in the CeA of CFA 4h *CaMKII-Cre* mice in the EPM (left, CFA 4h-EYFP, n = 7 mice; CFA 4h-eNpHR3.0-EYFP, n = 8 mice; time × group interaction, $F_{2,26} = 4.815$, $p = 0.0166$), OFT (middle, time × group interaction, $F_{2,26} = 3.511$, $p = 0.0447$), and pain threshold (right, time × group interaction, $F_{2,26} = 5.063$, $p = 0.0146$). Significance was assessed by a two-tailed paired Student's t test in **B**, two-way RM ANOVA with pre vs post *post hoc* comparison between groups in **C**, **E**, and **F**. The data are expressed as the mean ± SD. *$p < 0.05$; **$p < 0.01$; ***$p < 0.001$. ns, no significance.

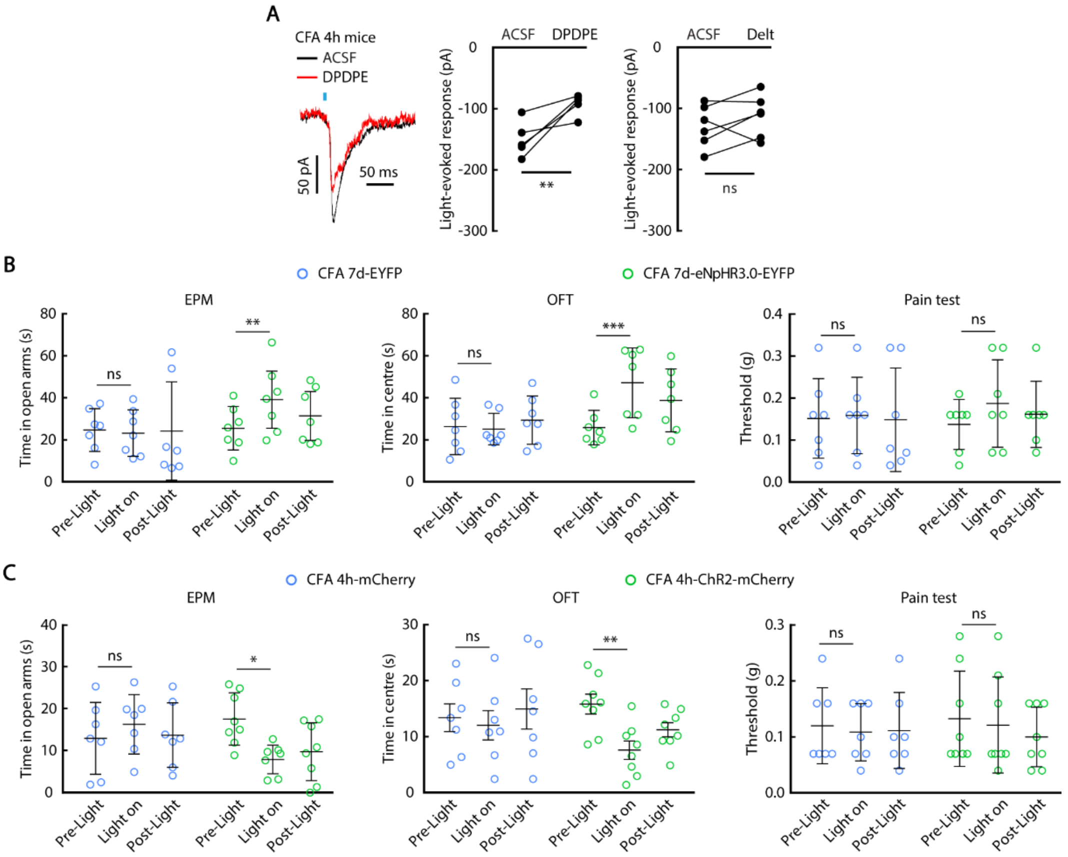

Figure S3. Optical inhibiton of PBN-CeA pathway reduces the anxiety of CFA 7d mice. (**A**) Sample traces and statistical data showing the effect of DPDPE ($n = 5$ cells from three mice; $t_4 = 3.723$, $p = 0.0204$) or deltorphin II ($n = 6$ cells from three mice; $t_5 = 1.323$, $p = 0.243$) on the light-evoked EPSCs following optical activation of the PBN^{Glu} terminals in the CeA from CFA 4h mice. (**B**) Summarized data showing the effect of optical inhibiton of PBN^{Glu} terminals in the CeA of CFA 7d *CaMKII-Cre* mice in the EPM (left, $n = 7$ mice each group; time × group interaction, $F_{2,24} = 3.773$, $p = 0.0376$), OFT (middle, time × group interaction, $F_{2,24} = 8.883$, $p = 0.0013$) and pain threshold (right, time × group interaction, $F_{2,24} = 0.4175$, $p = 0.6634$). (**C**) Summarized data showing the effect of optical activation of PBN^{Glu} terminals in the CeA of CFA 4h *CaMKII-Cre* mice in the EPM (left, CFA 4h-mCherry, $n = 7$ mice; CFA 4h-ChR2-mCherry, $n = 8$ mice; time × group interaction, $F_{2,26} = 3.878$, $p = 0.0336$), OFT (middle, time × group interaction, $F_{2,26} = 3.394$, $p = 0.049$), and pain threshold (right, time × group interaction, $F_{2,26} = 0.3935$, $p = 0.6787$). Significance was assessed by a two-tailed paired Student's *t* test in **A**, two-way RM ANOVA with pre vs post *post hoc* comparison between groups in **B**, and **C**. The data are expressed as the mean ± SD. *$p < 0.05$; **$p < 0.01$. ns, no significance.

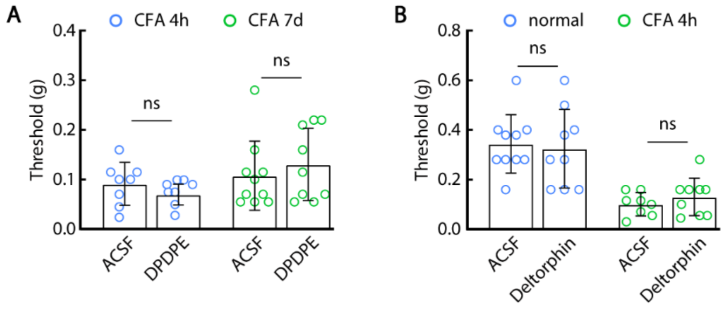

Figure S4. Effects of DPDPE and deltorphin II on pain threshold of normal, CFA 4h, and CFA 7d mice.
(**A**) Pain threshold of CFA 4h mice (n = 8 mice each group; t_{14} = 1.587, p = 0.1347) and CFA 7d mice (ACSF, n = 10 mice; DPDPE, n = 9 mice; t_{17} = 0.7058, p = 0.4899) following the CeA injection of ACSF and DPDPE. (**B**) Pain threshold of normal mice (ACSF, n = 10 mice; Deltorphin II, n = 9 mice; t_{17} = 0.3077, p = 0.7621) and CFA 4h mice (ACSF, n = 8 mice; Deltorphin II, n = 9 mice; t_{15} = 0.9664, p = 0.3492) following the CeA injection of ACSF and deltorphin II. Significance was assessed by a two-tailed paired Student's t test. The data are expressed as the mean ± SD. ns, no significance.
